# Safety of immune checkpoint inhibitors for cancer treatment: real-world retrospective data analysis from Qatar (SAFE-ICI-Q study)

**DOI:** 10.3389/fimmu.2025.1665716

**Published:** 2025-10-29

**Authors:** Nabil E. Omar, Shereen Elazzazy, Anas Hamad, Mohamed Omar Saad, Aya Alasmar, Sahar M. Nasser, Maria Benkhadra, Hebatalla M. Afifi, Farah I. Jibril, Rawan A. Dawoud, Mohamed S. Hamid, Afnan Alnajjar, Arwa O. Sahal, Amaal Gulied, Hazem Elewa

**Affiliations:** 1 Pharmacy Department, National Center for Cancer Care and Research, Hamad Medical Corporation, Doha, Qatar; 2 Clinical and Population Health Research Program, College of Pharmacy, QU Health, Qatar University, Doha, Qatar; 3 College of Pharmacy, QU Health, Qatar University, Doha, Qatar; 4 Pharmacy Department, Al Wakra Hospital, Hamad Medical Corporation, Doha, Qatar; 5 Medical Oncology Department, National Center for Cancer Care and Research, Hamad Medical Corporation, Doha, Qatar

**Keywords:** immune checkpoint inhibitors, immune-related adverse events, real-world data, cancer immunotherapy, biomarkers, progression-free survival, overall survival

## Abstract

**Introduction:**

Immune checkpoint inhibitors (ICIs) have significantly improved the therapeutic landscape of multiple malignancies. It becomes critical to understand the incidence, profile, and consequences of immune-related adverse events (irAEs) within real-world populations.

**Aim:**

We aimed to assess the safety profile of ICIs in adult cancer patients at the National Center for Cancer Care and Research (NCCCR), Qatar, and explore the factors associated with irAEs, including the impact of irAEs on the survival outcomes.

**Methods:**

This retrospective study included adult cancer patients who received at least one dose of an ICI between January 1, 2015, and January 1, 2020. Data was collected from electronic health records and institutional adverse drug reaction (ADR) reporting systems. irAEs were graded using Common terminology criteria of adverse events, version 5 (CTCAE v5). Logistic regression analysis was used to evaluate factors associated with irAEs. Kaplan–Meier and landmark analysis assessed associations between irAEs and progression-free survival (PFS) and overall survival (OS).

Approvals were obtained from HMC IRB (MRC-01-20-251) and Qatar University IRB (073/2025-EM).

**Results:**

A total of 236 patients (median age 57 years, 72% male) were included. Most patients had advanced solid tumors, with thoracic malignancies being the most common.

Pembrolizumab was the predominant agent used. irAEs occurred in 55.9% of patients, with the most frequent side effects being endocrine (26.4%), dermatologic (13.5%), and hepatic (12.4%) toxicities. Sixteen patients (6.8%) experienced fatal irAEs, with pneumonitis being the most common cause of death.

The median time to onset of irAEs was 55 days (IQR 16‐129.5 days). Most events occurred in the acute phase (21–180 days post-treatment). Resolution rates of irAEs varied, with gastrointestinal irAEs resolving in 92% of cases, compared to 40% for hematological events. Pulmonary irAEs were associated with the highest rate of treatment discontinuation.

Factors associated with irAEs included a higher number of ICI treatment cycles (p=0.019), lower baseline and six-week platelet counts (p=0.015 and p=0.012, respectively), and elevated baseline TSH (p=0.048). In multivariable regression analysis, the only factor that remained statistically significant was the number of treatment cycles (*p* = 0.004).

Dermatologic irAEs were significantly more common among patients aged ≥65 years (17.9% vs. 7.1%, p=0.018). Patients with poor performance status (PS ≥ 2) experienced a significantly higher rate of cardiac irAEs compared to those with good PS (10.9% vs. 1.7%, p=0.036).

In the 30-day landmark analysis, patients who developed irAEs had significantly worse PFS (3.3 vs. 7.1 months, p=0.0085) and OS (4.37 vs. 9.0 months, p=0.0004) compared to those without irAEs. These finding were confirmed using adjusted landmark analysis where irAEs were associated with worse OS (HR 2.13, 95% CI 1.34–3.3, *P* = 0.001) and PFS (HR 1.88, 95% CI 1.22–2.87, *P* = 0.004). Additionally, time-dependent Cox regression also demonstrated worse OS (HR 1.86, 95% CI 1.23–2.79, *P* = 0.003) and PFS (HR 1.96, 95% CI 1.41–2.72, *P* = 0.001).

**Conclusion:**

In this real-world cohort, irAEs were frequent and clinically diverse. Using adjusted landmark analysis and time-dependent Cox regression, early-onset irAEs were associated with inferior survival in our cohort. Poor baseline PS was linked to an increased risk of cardiac irAEs. Older adults were at a higher risk of dermatological irAEs. Some factors such as higher number of ICI treatment cycles, thrombocytopenia and elevated TSH at baseline may aid in risk stratification. These findings reinforce the need for timely detection and multidisciplinary management of irAEs to optimize ICI safety and effectiveness.

## Introduction

The immune system remains a complex and evolving field in oncology, offering a unique opportunity to expand therapeutic strategies. Over the past decade, the emergence of immune checkpoint inhibitors (ICIs) has marked a paradigm shift in cancer treatment by harnessing the innate ability of the immune system to detect and destroy malignant cells (1).

Using the host’s immunity to treat cancers depends on the immune surveillance: the ability of the immune system to identify foreign neo-antigens and target them for obliteration (2). Immune checkpoint receptors as cytotoxic T-lymphocyte-associated protein 4 (CTLA4) and programmed cell death protein-1 and ligand (PD-1, PDL-1) are crucial for the physiological responses of the immune system (3). Checkpoint signaling triggers immune tolerance of T-cell activation to avoid self-immunity and the adverse effects of excessive inflammatory responses. Tumor cells apply several mechanisms to avoid demolition by the immune system (3, 4). Unlike conventional chemotherapy, which is often limited by cumulative toxicity and non-specific mechanisms, ICIs work by blocking the inhibitory pathways which restrict T-cell activation (5). This allows for sustained immune responses against tumor cells, leading to durable responses in selected patients (6). Cytotoxic CD8+ T cells are within the adaptive immune system and represent the most powerful effectors in the anti-cancer immune response, forming the backbone of cancer immunotherapy. These therapies rejuvenate dysfunctional T cells, including CD8+ T cells (7).

In 2010, the FDA approved the first CTLA-4 inhibitor for the treatment of metastatic melanoma (8), then, PD-1 inhibitors were approved for the treatment of melanoma, renal cell carcinoma (RCC) and non-small cell lung cancer (NSCLC). After approval, these immunotherapeutic agents became important parts of the treatment protocols against melanoma and NSCLC (9). Furthermore, early clinical trials ICIs have shown encouraging results (objective response rates [ORRs]) against many types of cancers (10).

Therefore the indications of ICIs expanded to include additional types of cancers: biliary tract cancer, cervical cancer, cutaneous squamous cell carcinoma, endometrial, esophageal cancers, primary mediastinal large b-cell lymphoma, Merkle cell carcinoma and high tumor mutational burden cancers (11).

The very mechanism of action behind the clinical benefit from ICIs can engender unwanted inflammatory side effects (12). These immune-related adverse events (irAEs) can range from mild to life-threatening and may require prompt recognition and management (13). As the immune response triggered by these drugs is not entirely tumor-specific it can impact any other organ (12, 14). Moreover, these irAEs can pose a significant burden to the health systems (15, 16).

Although the side effects of ICIs are generally fewer and better tolerated than those of conventional chemotherapeutic agents, ICIs can still cause irAEs, including dermatological manifestations (such as reticular or maculopapular erythematous rash, pruritus, and mucositis), gastrointestinal toxicity (diarrhea and colitis), hepatic toxicity (elevated liver enzymes and hepatitis), and endocrine disturbances involving the pituitary, adrenal, or thyroid glands (17, 18).

The management of these irAEs usually requires immunosuppression, most commonly with corticosteroids, but may also include other agents (17, 18). Upon the expansion of the use of ICIs in clinical practice, rare or very rare side-effects are being discovered including hematological, infectious, neurological, renal, neurological irAEs and many others (19–25).

The time of onset of irAEs is less predictable. They may occur soon after receiving the first dose or long after a course of treatment has ended (26, 27).

Identification of reliable predictors of risk for irAEs is critical in guiding clinical decisions and facilitating treatment personalization.

Compared with biomarkers for tumor response using ICIs, those for irAEs have been less thoroughly investigated and some of the reported biomarkers for irAEs overlap with those for tumor responses (28). Some of these factors include ethnicity, age, sex, smoking status, pretreatment performance status (PS), elevated C reactive protein (CRP) from baseline (29–31), low muscle attenuation, sarcopenia, body mass index (BMI) (32, 33), absolute lymphocyte count (ALC) or eosinophil (34) were independent factors for irAEs development. Other factors including number of cycles, neutrophil to lymphocyte ratio (NLR) and platelet-to-lymphocyte ratio (PLR) might be important (35–37).

By explaining how these factors can influence the safety profile of ICIs, practitioners could personify regimes, monitor patients with high-risk profiles, and set in motion proactive approaches to minimize the likelihood of irAEs.

While clinical trials have established the safety and efficacy of ICIs across various types of cancer, real-world data remains essential to assess how these therapies perform in routine practice, especially in populations not well represented in trials, such as the Middle East and North Africa region. Such data are critical to inform healthcare providers, policymakers, and institutions on optimizing use, anticipating toxicities, and managing complications in everyday settings.

This critical disparity means that a considerable body of evidence on the incidence, spectrum, risk factors, and management of irAEs is based on data that may not fully reflect the unique characteristics of this geographically and ethnically distinct population.

Our study addresses this significant gap in the current literature by focusing on the real-world safety outcomes of ICIs in a patient population from the Middle East region. Our work aims to provide valuable, region-specific insights that can improve clinical decision-making.

At the National Center for Cancer Care and Research (NCCCR), the tertiary referral center for oncology in Qatar, ICIs have been widely adopted since their initial approval in 2011. In this context, we aim to retrospectively describe and evaluate the safety profile of ICIs. This includes characterizing the incidence, type, and management of irAEs, as well as identifying any demographic, laboratory, clinical or biochemical factors associated with their occurrence. Moreover, we aim to explore the association between the incidence of irAEs and survival outcomes.

## Method

This is a retrospective cohort study conducted at NCCCR, Hamad Medical Corporation (HMC), Qatar. NCCCR is the only tertiary hospital managing cancer in the state of Qatar. The cohort included adult cancer patients newly initiated on ICIs who received at least one dose between January 1, 2015, and January 1, 2020. ICIs include: pembrolizumab, nivolumab, atezolizumab, avelumab, durvalumab, or ipilimumab. Pediatric cases and patients receiving only other systemic anticancer therapies during the same period were excluded.

Patient data were retrospectively extracted from the electronic health record (Cerner^®^), medication administration records, progress and discharge summaries, and the institutional adverse drug reaction (ADR) reporting system. IrAEs were identified and graded according to the Common Terminology Criteria for Adverse Events (CTCAE) version 5.0. This study was conducted under institutional approval with a waiver of informed consent, given its retrospective design, use of existing medical records, and absence of direct patient contact or intervention. All data were anonymized prior to analysis, and confidentiality was strictly maintained in accordance with institutional and international ethical standards. Ethical approvals were obtained from the Hamad Medical Corporation IRB (MRC-01-20-251) and the Qatar University IRB (QU-IRB 073/2025-EM).

The study comprehensively captured variables including demographics (age, gender, ethnicity, smoking status, BMI, comorbidities), cancer type and stage, PS at baseline, prior lines of therapy, specific ICI administered (agent, dose, number of cycles), and baseline investigations (complete blood count, metabolic panel, thyroid function, echocardiography). Additionally, other immune biomarkers were recorded such ALC, eosinophil counts, NLR, PLR and CRP values at baseline and week 6 of ICIs administration.

For each irAEs, the type, time to onset, grade, and management (e.g., corticosteroids, intravenous Immunoglobulins (IVIG), infliximab or other agents) were documented, including treatment outcome, resolution or discontinuation of ICI due to irAEs.

We also collected data about rechallenge of ICIs post development of high grade irAEs and whether the side effects recur or not. Cause of death was identified by multidisciplinary team and verified by medical oncologist team member to be either cancer related, non-cancer related or fatal irAEs.

### Statistical analysis

Categorical variables were summarized as counts with corresponding percentages, while continuous variables were reported as medians with interquartile ranges (IQR). Comparisons between groups were performed using the chi-square test or Fisher’s exact test for categorical variables, as appropriate, and the Wilcoxon rank-sum test for continuous or ordinal variables. Age was dichotomized at 65 years to identify older adults, and PS was categorized according to the Eastern Cooperative Oncology Group (ECOG) into “Good” (ECOG 0–1) and “Poor” (ECOG 2–4) groups.

Based on current literature, we divided the time of onset of irAEs into 4 categories according to chronicity: hyperacute (less than 21 days after initiation) (38–42), acute (21 to less than 180 days) (43–45), late (180 to less than 365 days) (46), and delayed (above 365 days) (47–49).

Independent associations of baseline characteristics with irAEs were explored using multivariable logistic regression. We used backward elimination to exclude variables with p-values of 0.2 or more from the model. Candidate predictor variables included demographic factors (age, sex, BMI, PS and smoking status), comorbid conditions (diabetes mellitus, hypertension, chronic kidney disease, chronic liver disease, coronary artery disease, lung disease, dyslipidemia, and thyroid disorder), laboratory parameters (thyroid-stimulating hormone [TSH], NLR, and platelet count), and treatment-related variables (number of cycles received, place of therapy of ICIs {first, second or subsequent line of therapy}, type of ICIs used, and presence of ≥2 metastatic sites).

Time-to-event outcomes included overall survival (OS) and progression free survival (PFS). OS was defined as the time from treatment initiation to death. PFS was defined as the time from treatment initiation to either documented disease progression or death, whichever occurred first.

Follow-up times were censored at the date of last follow-up for patients who did not experience disease progression or death. Survival analyses were limited to each patient’s first treatment record to ensure independence of observations.

To account for potential immortal time bias and explore the associations of irAEs occurring at different time points, landmark analyses were conducted at 30-, 60-, 90-, 180-, and 360-days following treatment initiation. At each landmark, patients who had not yet experienced an outcome event were grouped according to whether an irAE had occurred before that time point. Survival was then analyzed from each landmark forward among these groups. Kaplan–Meier method was used to estimate survival distributions for both PFS and OS. Median survival times and corresponding 95% confidence intervals were calculated. Survival was compared between patients who experienced irAEs and those who did not, using the log-rank test. To account for confounding, we conducted adjusted landmark analyses using multivariable Cox regression models. To assess the robustness of the findings from the landmark analyses, we also conducted multivariable Cox regression of OS and PFS in which irAE was modelled as a time-dependent variable. All Cox regression models included the following covariates: age, PS, presence of ≥2 metastatic sites, diabetes mellitus, hypertension, chronic kidney disease, chronic liver disease, coronary artery disease, lung disease, and cancer type.

All statistical tests were two-sided, and p-values of <0.05 were considered statistically significance. Analyses were conducted using Stata version 17 (StataCorp, College Station, TX, USA).

## Results

A total of 236 cancer patients received ICIs at NCCCR during the study period (2015 – 2020). Among the 236 patients, a total of 249 ICI treatment regimens were administered, accounting for patients who received sequential therapy with different ICIs (13 patients were rechallenged with a different ICI after disease progression on a prior one). The baseline characteristics of the patients are presented in (**Table 1**). The median age was 57 years (47–66 years), and male gender comprised up to 72% of the population, the male/female ratio was 2.5:1.

**Table 1:** Baseline characteristics of included patients grouped by the incidence of irAEs:.

Category				P value
	Total patients n (%)	Patients with irAEs n (%)	Patient without irAEs n (%)	
Number of patients	236	132	104	
Age (median, IQR)	57 (47-66)	59 (46-66)	56 (50-63)	0.73
Male	169 (71.6%)	91 (68.9%)	78 (75%)	
WHO Regional Grouping				0.53
African Region	5 (2.10%)	3 (2.3%)	2 (1.9%)	
Eastern Mediterranean Region	164 (69.5%)	97 (73.5%)	67 (64.4%)	
European Region	7 (3%)	4 (3%)	3 (2.9%)	
Americas	8 (3.4%)	3 (2.3%)	5 (4.8%)	
South-East Asia Region	34 (14.4%)	18 (13.6%)	16 (15.4%)	
Western Pacific Region	18 (7.6%)	7 (5.3%)	11 (10.6%)	
Baseline co-morbidities				
Diabetes Mellitus (DM)	77 (32.6%)	48 (36.4%)	29 (27.9%)	0.17
Hypertension (HTN)	95 (40.3%)	56 (42.4%)	39 (37.5%)	0.44
Chronic Kidney Disease (CKD)	19 (8.1%)	10 (7.6%)	9 (8.7%)	0.76
Chronic Liver Disease (CLD)	5 (2.1%)	4 (3%)	1 (1%)	0.39
Coronary Artery Disease (CAD)	18 (7.6%)	11 (8.3%)	7 (6.7%)	0.65
History of Baseline Autoimmune Disease	8 (3.4%)	6 (2.5%)	2 (0.9%)	0.57
Smoking Status				0.33
Smokers	85 (36%)	44 (33.3%)	41 (39.4%)	
Non-smokers	151 (64%)	88 (66.7%)	63 (60.6%)	
Baseline BMI *(kg/m²)*	25.0 (21.9-29.1)	25.3 (21.9-30)	24.8 (21.8-28.3)	0.28
≥ 30	51 (21.6%)	33 (25%)	18 (17.3%)	0.15
< 30	185 (78.4%)	99 (75%)	86 (82.7%)	
PD-L1 Expression				0.97
> 50%	17 (7.20%)	7 (5.3%)	7 (6.7%)	
1-49%	14 (5.90%)	4 (3.0%)	5 (4.8%)	
<1%	9 (3.80%)	10 (7.6%)	7 (6.7%)	
CPS >1	5 (2.1%)	3 (2.3%)	2 (1.9%)	
CPS >20	2 (0.8%)	1 (0.8%)	1 (1.0%)	
Not Done	189 (80.1%)	107 (81.1%)	82 (78.8%)	
Baseline Performance Status				0.72
Good (0-1)	172 (72.90%)	95 (72.0%)	77 (74.0%)	
Poor (2-4)	64 (27.10%)	37 (28.0%)	27 (26.0%)	
Number of Metastases				0.1
0	18 (7.6%)	13 (9.8%)	5 (4.8%)	
1 Site	80 (33.9%)	48 (36.4%)	32 (30.8%)	
2 or more site	138 (58.5%)	71 (53.8%)	67 (64.4%)	
Metastases sites				
Liver	63 (26.7%)	36 (27.3%)	27 (26.0%)	0.82
Lung	88 (37.3%)	44 (33.3%)	44 (42.3%)	0.16
Bone	83 (35.2%)	44 (33.3%)	39 (37.5%)	0.51
Lymph Nodes	129 (54.7%)	69 (52.3%)	60 (57.7%)	0.41
CNS	32 (13.6%)	17 (12.9%)	15 (14.4%)	0.73
Number of ICI Cycles	5 (2-10)	6.0 (2.5-15.0)	4.5 (2.0-7.0)	0.019
Duration week	12 (4-31)	12.1 (5.6-41.9)	10.9 (3.1-21.0)	0.083

WHO, World Health Organization; BMI, Body Mass Index; PD-L1, Programmed Death-Ligand 1; CPS, Combined Positive Score; CNS, Central Nervous System; ICI, Immune Checkpoint Inhibitors.

**Figure 1:**
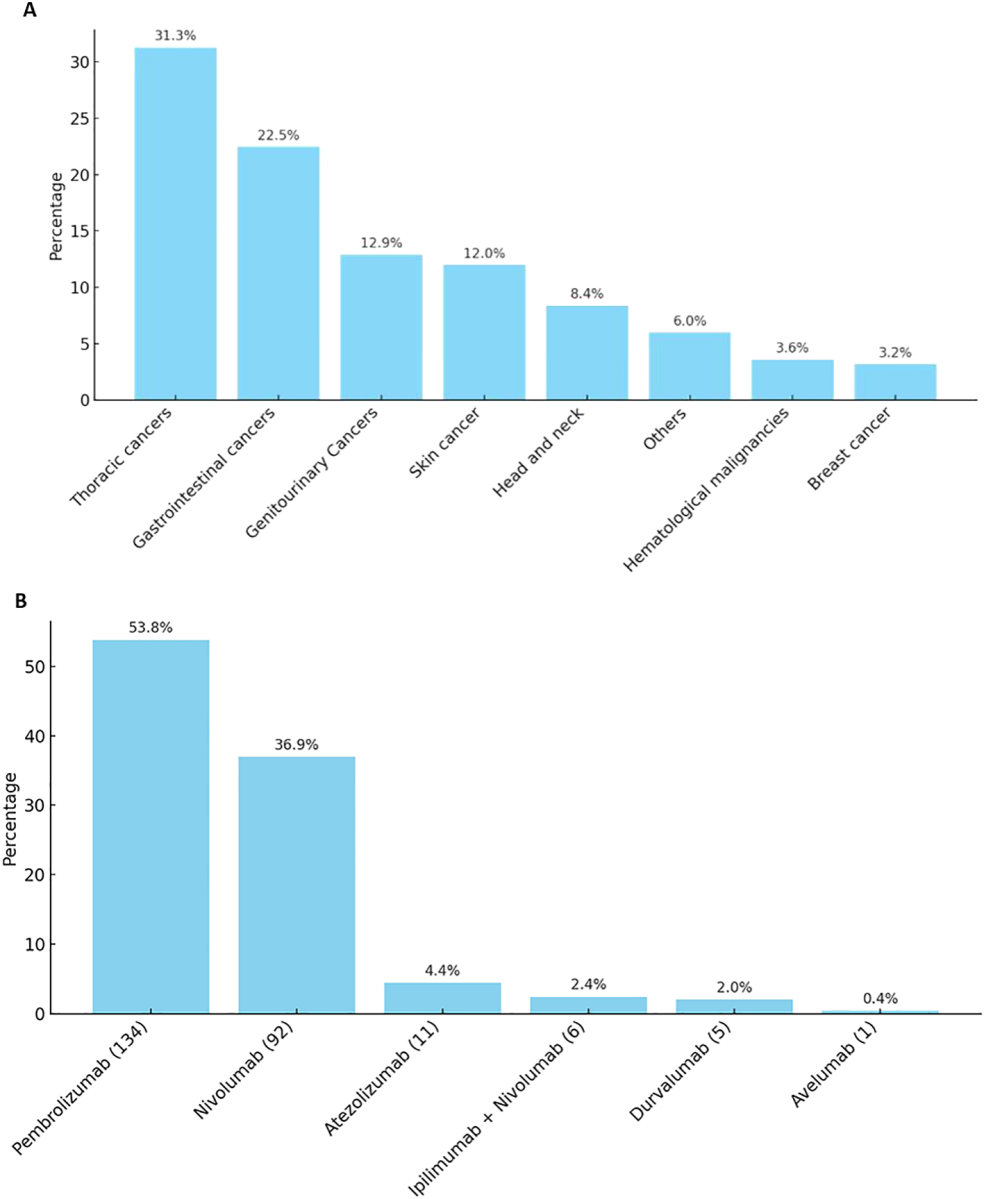
**(A)**. Patients who received immunotherapy distribution by diagnosis. **(B)**. Distribution of ICIs agent received.

Most of the patients were of eastern mediterranean descent (69.5%), while the least was of African descent (2.1%). Obese patients (BMI ≥ 30) represent 21.6%. Most of the patients (72.90%) had good PS. More than 90% of the patients had at least 1 comorbidity, the majority of which were hypertension (HTN; 40%) followed by type 2 diabetes mellitus (33%). Patients who had history of baseline autoimmune disease represented 3.4%. Most of the patients were diagnosed with solid tumors and predominance of thoracic malignancy followed by gastrointestinal, genitourinary and skin cancer respectively (**Figure 1A**). Pembrolizumab was the most frequently used agent (53.8%) (**Figure 1B**).

The majority of ICIs were initiated in the second-line treatment setting (**Supplementary Figure S1**).

Most patients (92%) had metastatic disease (> 1 site of metastases), and liver metastasis was associated with numerically increased risk of irAEs, however not statistically significant (p=0.82).

The median duration of treatment was 12 weeks (IQR 4 to 31 weeks). The median number of treatment cycles received was 5 cycles (IQR 2 to 10 cycles). Higher number of ICIs cycles was associated with an increased occurrence of irAEs (p=0.019).

**Figure 2:**
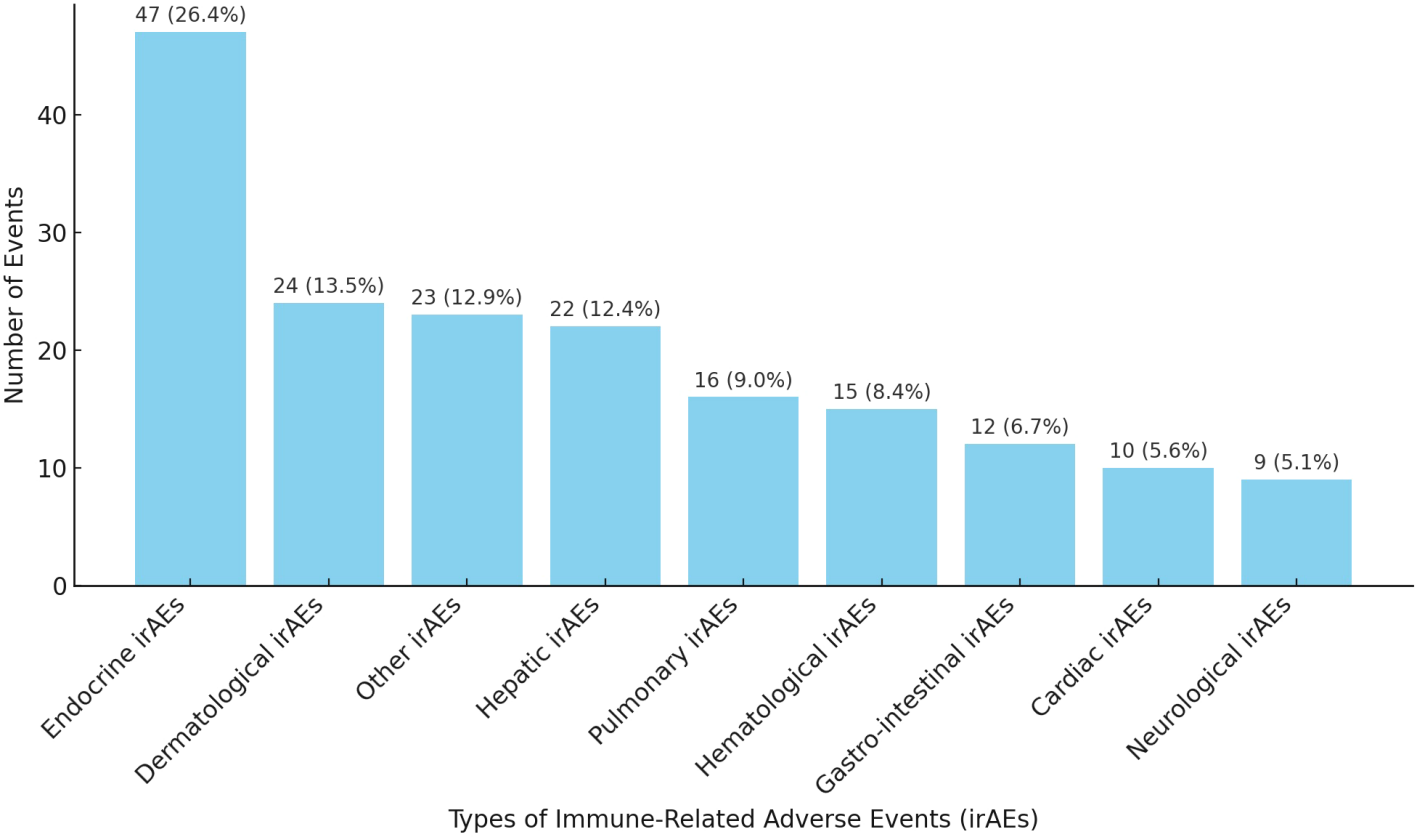
Distribution of irAEs per system/organ. *Other irAEs: Musculoskeletal, Renal, Infusion reaction, Infectious and Ocular irAEs.

Out of the total number of patients (236 patients) receiving ICIs, 132 (55.9%) developed irAEs, with a total of 178 events reported. Immune related endocrinopathies were the most common irAEs (26.4%) followed by dermatological toxicities (13.5%) (**Figure 2**). The descriptions of irAEs per organ/system affected are detailed in **Supplementary Materials** (**Supplementary Table S1**).

The discontinuation rate of ICIs post-irAEs was highest due to pulmonary irAEs which is consistent with the pulmonary toxicity being the third highest among grade 4 irAEs.

When patients were rechallenged with ICI after irAEs, the recurrence rates for hepatic, dermatologic, and endocrinological irAEs were 9%, 4%, and 2%, respectively.

**Figure 3:**
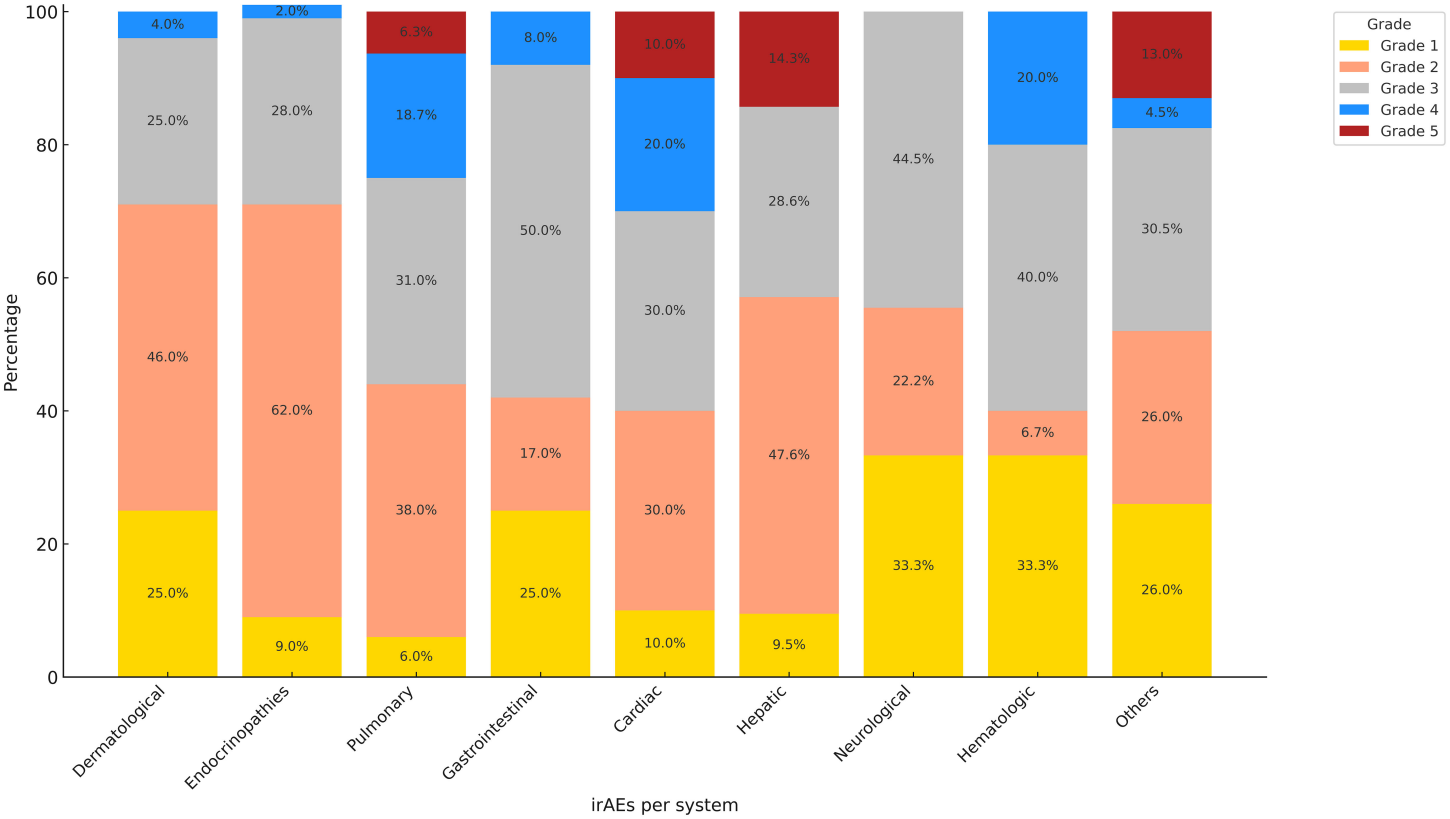
Grading of irAEs according to CTCAE criteria (%).

The irAEs ranged from grade 1 to grade 5 toxicities according to CTCAE Version 5 criteria, the most common grade 4 toxicities were both cardiovascular (20%) and hematological toxicities (20%) followed by pulmonary toxicity (18.7%) (**Figure 3**).

The median time of onset for irAEs was 55 days (IQR 16‐129.5 days). However, we noticed 3 cases of infusion-related reactions occurred 10 minutes after the first infusion of ICI monotherapy (**Supplementary Table S2**).

**Figure 4:**
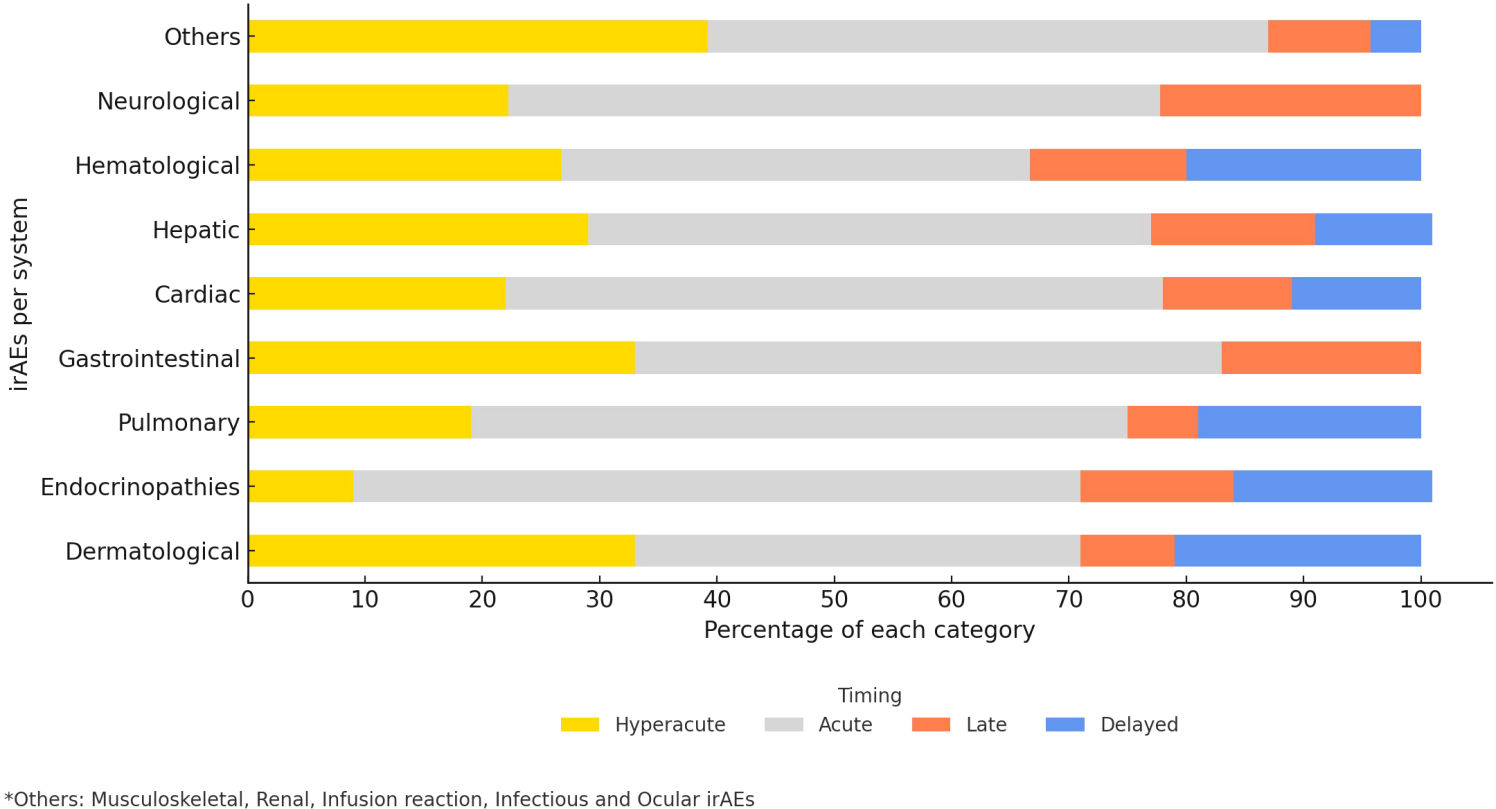
Time of onset of irAEs (%).

In this study, 55.1% of the observed irAEs occurred acutely (within 21 to less than 180 days from ICIs initiation), with endocrinopathies (62%) being the most common acute events followed by pulmonary (56%), cardiovascular (56%), and neurological (55.6%) adverse events (**Figure 4**). Among the hyperacute complications, dermatological (33%) as well as gastrointestinal (33%) adverse events were the most common. Delayed irAEs accounted for 8.8% of the total number of irAEs. Dermatological (21%), hematological (20%) and pulmonary events (19%) were amongst the most common delayed irAEs, gastrointestinal or neurological events were not observed amongst this category.

Most of the irAEs were resolved post proper management, with gastrointestinal irAEs achieving the highest percentage of resolution (92%) while the least were hematologic irAEs (40%) as shown in (**Table 2**).

**Table 2:** Outcome characteristics of irAEs.

	Number of irAEs*	Outcomes of irAEs¥			
System	Total n= 178 (100%)	irAEs resolvedn (%)	ICI discontinued due to irAEsn (%)	Rechallenge of ICI after irAEsn (%)	Recurrence of irAEsn (%)
Dermatological	24 (13.5)	18 (75)	5 (21)	3 (13)	1 (4)
Endocrinopathy	47 (26.4)	32 (68)	5 (10.6 )	4 (8.5)	1 (2)
Pulmonary	16 (9)	8 (50)	11 (69)	2 (12.5)	0
Gastrointestinal	12 (6.7)	11 (92)	3 (25)	3 (25)	0
Cardiovascular	10 (5.6)	4 (40)	6 (60)	1 (10)	0
Hepatic	22 (12.4)	11 (50)	11 (50)	2 (9)	2 (9)
Hematological	15 (8.4)	6 (40)	5 (33.3)	2 (13.3)	0
Neurological	9 (5.1)	5 (55.6)	3 (33.3)	0	0
Other#	23 (12.9)	12 (52.2)	8 (34.8)	4 (17.4)	0

* Percentage calculated from total number of irAEs (N=178).

¥ Percentage calculated from each corresponding irAEs.

# Musculoskeletal, Renal, Infusion reaction, Infectious and Ocular irAEs.

We summarized treatments by organ system as shown on supplementary (**Supplementary Table S3**).

In our cohort, irAEs management was predominantly non-immunosuppressive (122 episodes), especially for endocrine (n=42) and skin (n=17) events. Non-immunosuppressive management strategies were frequently employed across different organ systems. In endocrine irAEs, hormone replacement or disease-specific therapy was the mainstay: levothyroxine for hypothyroidism, insulin or oral hypoglycemics for diabetes, carbimazole for hyperthyroidism, and fluid restriction for hyponatremia or SIADH. Dermatologic events were often controlled without the need for systemic corticosteroids, using supportive measures such as topical corticosteroids with or without antihistamines, oral antihistamines, emollients, or antipruritic agents like hydroxyzine. Infectious complications were managed with supportive therapy, including intravenous fluids, antibiotics, or anti-tubercular medications as indicated. Blood transfusion and/or IVIG were used to treat some hematological irAEs. Renal irAEs were typically addressed with aggressive hydration, supportive fluid therapy, or renal replacement therapy in some cases. Musculoskeletal events such as fatigue were successfully managed with analgesics or physiotherapy. Moreover, most low-grade irAEs were resolved simply by withholding ICIs under close monitoring.

Systemic corticosteroids were used in 49 episodes, most commonly for pulmonary (n=10), hepatic (n=7) and skin (n=7) irAEs, with fewer for endocrine (n=5), cardiac (n=4), neurologic (n=3), GI (n=2), and hematologic (n=1). Second-line immunosuppressants were infrequent (n=12) and largely confined to hepatic irAEs (n=9).

### Fatal irAEs

Sixteen patients (6.8%) developed 20 fatal irAEs, accounting for 11.2% of the total irAEs reported. Most of these patients were male, younger than 65 years old with poor PS and were having stage 4 at the time of their initial diagnosis (**Table 3**). The most used agent was pembrolizumab. The most common fatal irAEs was pneumonitis (n=5; 25%) with median time to death of 6 days. Cardiac, hepatic, and renal systems were equally affected (n=4; 20%) with median time to death of 5.7, 20.4, and 18.8 days respectively. Fatal endocrine, nervous system and hematological adverse events were each detected once (5%) with median time to death of approximately 9 days (**Supplementary Table S4**). Seventy percent of the fatal irAEs occurred within the first 21 days of ICI therapy initiation (**Supplementary Table S5**).

**Table 3:** Characteristics of Patients Who Developed Fatal irAEs (n=16).

Characteristic	n (%)
Age ≥ 65	6 (37.5)
Male Gender	9 (56.25)
Poor PS ECOG ≥ 2	9 (56.3)
Diagnosis	
Gastrointestinal cancers	4 (25)
Thoracic cancers	4 (25)
Genitourinary cancers	3 (18.7)
Others (Breast, head and neck, endometrial, and unknown primary cancers)	5 (31.3)
Stage IV	11 (68.75)
Pembrolizumab	8 (50)
Nivolumab	5 (31.3)
Others (Atezolizumab, Durvalumab, and Nivolumab Ipilimumab combination)	3 (18.7)

### Factors associated with irAEs

In the univariate analysis of clinical, laboratory, and biochemical parameters associated with irAEs occurrence, several associations were observed (**Table 4**). A higher number of treatment cycles was associated with an increased incidence of irAEs (p = 0.019).

**Table 4:** Univariate analysis of clinical, laboratory and biochemical parameters associated with irAEs occurrence.

Patients’ factor	Y (irAEs occurrence)	N (no irAEs)	P-value
Clinical Parameters			
Number of cycles	6.0 (2.5–15.0)	4.5 (2.0–7.0)	0.019
Laboratory parameters			
Baseline Platelets (PLT) (×10^9^;/L)	233.0 (175.5–300.0)	277.0 (205.5–343.5)	0.015
PLT at 6 weeks (PLT W6)	222.5 (158.0–320.0)	258.0 (195.0–358.0)	0.012
Biochemical Parameters			
Baseline TSH (mIU/L)	1.9 (1.0–3.4)	1.6 (0.8–2.6)	0.048

Among laboratory parameters, lower platelet counts at baseline and at six weeks intervals (p = 0.015 and 0.012, respectively) were associated with a higher incidence of irAEs. For the biochemical factor, higher baseline TSH levels (p = 0.048) were also associated with increased risk of irAEs.

Other clinical, laboratory, or biochemical factors, including age, gender, BMI, smoking status, PS, cancer type, absolute neutrophil count (ANC), eosinophil count, ALC, NLR and PLR did not show significant differences between patients with and without irAEs.

In the multivariable logistic regression model, only the number of cycles remained statistically significant (p=0.004).

### Association between irAEs and survival outcomes

Using unadjusted landmark analysis at 30, 60, 90,180 and 360 days, we explored the association between irAEs and survival outcomes (PFS and OS). No statistically significant association was observed at any of the landmark time points (**Supplementary Figures S2A, B**) except at 30-days mark.

**Figure 5:**
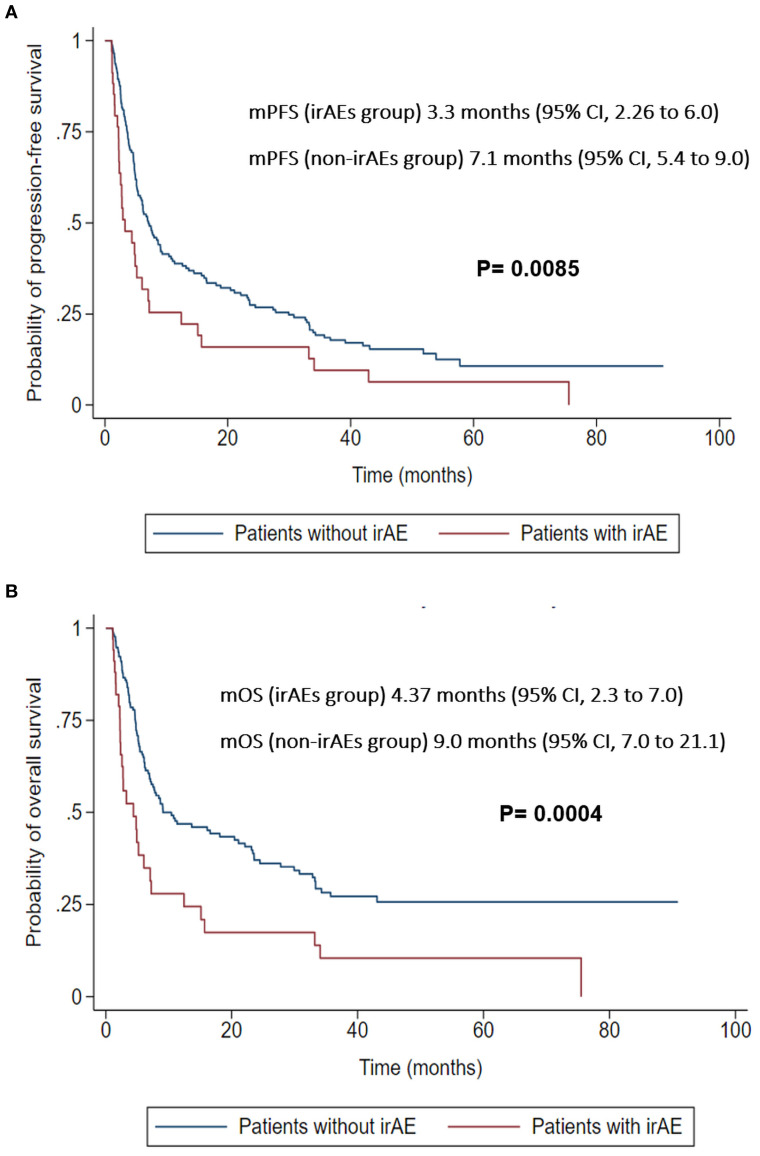
**(A)** Landmark analysis of PFS at 30 days. **(B)** Landmark analysis of OS at 30 days.

At the 30-days landmark, patients who developed irAEs had worse median PFS (mPFS) of 3.3 months (95% CI, 2.26 to 6.0) compared to those without irAEs, who had a mPFS of 7.1 months (95% CI, 5.4 to 9.0) (P = 0.0085) (**Figure 5A**).

Similarly, at 30-days landmark, patients who developed irAEs had worse median OS (mOS) of 4.37 months (95% CI, 2.3 to 7.0) compared to those without irAEs, who had a mOS of 9.0 months (95% CI, 7.0 to 21.1) (P = 0.0004) (**Figure 5B**).

In the adjusted landmark analysis, patients who developed irAEs within the 30-day landmark demonstrated significantly worse overall survival (OS; HR 2.13, 95% CI 1.34–3.30, *P* = 0.001) and progression-free survival (PFS; HR 1.88, 95% CI 1.22–2.87, *P* = 0.004). No statistically significant associations were observed at the other tested landmarks, as detailed in **Supplementary Table S6**.

Time-dependent Cox analysis confirmed this association, demonstrating an independent association of irAEs with worse OS (HR 1.86, 95% CI 1.23–2.79, *P* = 0.003) and PFS (HR 1.96, 95% CI 1.41–2.72, *P* = 0.001).

### irAEs and age

Among the 236 patients, 169 were younger adults (below 65 years), and 67 patients were older adults (65 years and above). Among younger adults, 90 patients (53.3%) developed irAEs compared to 42 patients (62.7%) in the older adult group (p=0.19). The incidence of cardiac, hepatic, pulmonary, endocrine, gastrointestinal and miscellaneous toxicities was similar between the two groups, while dermatologic toxicity was significantly higher in older adults (17.9% vs. 7.1%, p = 0.018).

### irAEs and PS

Of the 236 patients evaluated, 172 (72.9%) had good PS, while 64 (27.1%) had poor PS. The incidence of irAEs was similar between the two groups: 95 patients (55.2%) in the good PS group and 37 patients (57.8%) in the poor PS group (p = 0.77). However, cardiac irAEs were significantly more frequent in the poor PS cohort, occurring in 10.9% compared to 1.7% in the good PS group (p = 0.036).

## Discussion

Our study provides one of the largest real-world analyses of ICI safety outcomes in a cohort from the Middle East. The findings on the incidence and spectrum of irAEs offer a crucial perspective that complements the data from pivotal clinical trials, which often lack representation from this population.

By highlighting the unique regional characteristics of irAEs, our results serve as a vital contribution to the global ICI safety literature. This real-world evidence can help guide clinicians in managing toxicities and optimize patient care in similar underrepresented populations worldwide.

In our cohort, we identified 236 patients who received ICIs at NCCCR representing real-world insights into the incidence and clinical profile of irAEs, risk factors, and clinical outcomes. These results align with existing literature and provide additional data on the frequency, severity, and timing of irAEs.

Firstly, the overall incidence of irAEs in this cohort was 55.9%, comparable to the 40–60% range seen in clinical trials (13, 50), but higher than that reported in real-world studies showing a 30–43% incidence (51, 52). In our study, there were no statistically significant differences in incidence of irAEs based on age, ethnicity, gender, BMI, smoking status, or cancer type.

Literature showed that endocrine, dermatologic, and gastrointestinal irAEs are the most commonly reported (53, 54). Consistent with global data, endocrinopathies were the most common toxicity (26.5% in our study vs. 20 to 35% in the literature) (12, 55). Dermatologic and hematologic toxicities occurred in 13.5% and 5.5% (20) of our patients, respectively, which is consistent with previous reports ranging from 10 to 15% (55, 56). However, gastrointestinal toxicities occurred in 7% of our patients, lower than the 26.3% reported in a prior meta-analysis (12), which could be due to under-documentation in our cohort.

We reported grade 4 cardiovascular (20%) and hematologic toxicities (20%), which were 2 to 3 times higher than those reported in trials, ranging from 5 to 10% (57). This may be attributed to underreporting, or misclassification in trials, and the more complex nature of real-world patients.

In randomized clinical trials, the incidence of serious and fatal irAEs is frequently underestimated, partly due to the limited availability of comprehensive and reliable data (58).

While our study demonstrated a high rate of fatal irAEs in real-world setting. In our cohort, 6.8% of patients developed 20 fatal irAEs (11.2% of all irAEs), thereby surpassing the 0.3–1.3% reported in large meta-analyses and other studies (13, 59, 60). This may reflect our population’s high-risk demographics: 92% had metastatic disease, 27% had poor PS (ECOG ≥2), 40% had hypertension, and 33% had diabetes.

The most common fatal irAEs in our study was pneumonitis, highlighting the aggressive nature of certain irAEs. Fatal cardiac, hepatic, and renal toxicities each occurred in one-fifth of the patients who developed fatal irAEs. These findings are consistent with those of a large meta-analysis, which identified pneumonitis (35%) as the leading cause of fatal irAEs, followed by myocarditis (25%) and hepatitis (22%) (13).

Furthermore, in our study, we stratified the chronicity irAEs onset into hyperacute, acute, late, and delayed phases. The overall median time to onset of all grade irAEs was 55 days (IQR 16–129.5), consistent with the 6–12 weeks window described in the literature (61). Moreover, we found that 55.1% of irAEs occurred acutely (21–180 days), while 70% of the fatal irAEs occurred hyper-acutely (<21 days), emphasizing the importance of early detection.

Delayed irAEs occurred in 8.8% of cases, exceeding the earlier reports of <5%, and reflecting increased recognition of long-term toxicities (47, 62, 63).

Very early or hyperacute irAEs such as fulminant myocarditis, pneumonitis and, nephritis have been documented within the first 2–3 weeks of ICI therapy (38–40). Other reports showed that myocarditis associated with ICIs usually occur early, Johnson et al. reported a median onset of 17 days (41), while a larger registry analysis by Moslehi et al. observed a similar pattern, with a median onset of 27 days after treatment initiation (42). Capturing this window is clinically important because such early toxicities often represent fulminant, high-grade events requiring urgent intervention.

The time frame of 3-week to 6-months window corresponds to the typical period of maximal immune activation when checkpoint blockade is exerting its intended anti-timer effects and also when the majority of collateral autoimmune effects which are translated as acute irAEs. ESMO Clinical Practice Guideline showed that the majority of irAEs emerge within the first 8–12 weeks of treatment initiation, with dermatologic toxicities frequently being the earliest to appear (43).

The ASCO guideline further endorsed similar time frame, noting that most irAEs may arise up to 26 weeks, with a median onset of approximately 40 days (44). Moreover, A pooled analysis of 23 clinical trials and 8,436 patients demonstrated that the median onset for most irAEs ranges from 2–15 weeks, with some extending toward 6 months (45).

Real-world cohorts demonstrated that a subset of patients (5–10%) experience their first irAEs in the late window, typically between 6 and 12 months after initiating immunotherapy (46).

Moreover, delayed irAEs (DIRE), defined as toxicities occurring more than one year after initiation or several months following discontinuation of ICIs, have been consistently documented in long-term follow-up studies and were endorsed by the Society for Immunotherapy of Cancer (SITC), as a distinct clinical entity (47–49).

Notably, higher cycle numbers were significantly associated with an increased irAEs risk (p=0.019), while longer treatment duration had a non-significantly higher trend with irAEs risk (p=0.083). This was consistent with the findings from Weber et al., that reported a 26% increase in irAEs risk per additional ICI cycle (64).

Yao and colleagues systematically analyzed clinical and translational data to identify determinants of tumor response and predictors of irAEs in patients receiving ICIs (65). They found that cytokine profiles, particularly elevated baseline or early increases in pro-inflammatory mediators such as IL-6, IL-17, IFN-γ, and TNF-α, may serve as early biomarkers of severe irAEs.

In addition, T-cell receptor (TCR) repertoire dynamics were linked to improved tumor control but also greater susceptibility to autoimmune toxicities, indicating that the same immune mechanisms contribute to both efficacy and adverse events. Furthermore, baseline autoimmune markers, including pre-existing autoantibodies such as antinuclear antibodies (ANA) and thyroid antibodies, were identified as predictors of irAEs. Collectively, these findings provide a mechanistic basis for integrating immune biomarkers into early detection and monitoring strategies, facilitating risk stratification and proactive irAEs management while preserving therapeutic benefit.

Among biomarkers that have emerged as predictors of irAEs include eosinophilia, CRP, NLR, PLR, and ALC (31, 34–36, 66). Studies reviewing these correlations have shown conflicting results. Eosinophilia was significantly associated with the development of irAEs, particularly endocrine-related events (67). Similarly, elevated CRP levels measured before and during treatment were linked with a higher risk of irAEs incidence and worse overall prognosis (68, 69).

In addition, both high NLR and PLR have been shown to predict the risk and severity of irAEs. Specifically, an NLR >4.3 or PLR >210 was associated with significantly worse outcomes and a higher likelihood of high-grade irAEs (70–72).

Furthermore, high ALC above 820 cells/µL measured two weeks after initiating ICIs was predictive of early onset irAEs (73). On the other hand, several studies have demonstrated a neutral or even protective association between ALC, NLR, or PLR and the incidence of irAEs. Nevertheless, there is no universal consensus on the optimal predictive cut-off values for these biomarkers (66).

In line with these findings, our study did not identify any statistically significant association between eosinophil count, ALC, NLR or PLR and irAEs incidence.

The likelihood of experiencing irAEs may also depend on the type of cancer. irAEs were more prevalent in patients with NSCLC, RCC, and melanoma (74).

Recent evidence suggests that certain genitourinary (GU) malignancies, particularly RCC, may exhibit a distinct irAEs profile. In a large retrospective cohort, patients with RCC had significantly higher odds of developing any irAEs (adjusted OR = 1.8) compared to those with non-GU cancers. In contrast, urothelial carcinoma patients in the same cohort showed similar irAEs profile without a statistically significant increase in overall irAEs risk or unique organ-specific toxicities compared to non-GU cancers (75).

Moreover, in RCC patients receiving a combination of ICIs and anti-angiogenic agents, certain irAEs, such as hematologic toxicities, appeared less frequent, whereas others, particularly hepatitis and adrenal insufficiency, were more commonly observed (76).

Some cancer-type-specific immunopathological mechanisms have been proposed. Immunologically “hot” tumors such as RCC demonstrate stronger responses to ICIs (65, 74), but this robust immune activation also increases the risk of collateral irAEs compared to “cold” tumors like prostate cancer (75). Distinct irAEs patterns are further influenced by treatment context (77, 78), as dual checkpoint blockade (PD-1 plus CTLA-4, IO–IO) is frequently used in RCC but not established as standard for other GU cancers such as bladder cancer, where guidelines recommend PD-1/PD-L1 monotherapy (e.g., pembrolizumab, avelumab maintenance) due to excess toxicity without proven survival benefit. The use of dual IO–IO is known to amplify toxicity, potentially explaining the higher rates of myocarditis and multi-organ irAEs in RCC cohorts (75). Although the precise mechanisms remain under investigation, emerging evidence suggests that while “on-target” immune activation drives anti-tumor efficacy, the resulting “off-target” effects are shaped by tumor immunogenicity and cancer type.

Higher BMI has been associated with a greater likelihood of developing irAEs. A comprehensive meta-analysis revealed that obese patients had significantly increased odds of experiencing irAEs (OR 2.62, 95% CI 1.70–4.03) (79). However, this association was not observed in our cohort.

Racial disparities and ethnic backgrounds may influence the incidence of irAEs and survival outcomes. Several studies have shown that Caucasians had higher rates of irAEs and better OS compared to African Americans and Hispanics (80, 81). Another study showed that despite a similar irAEs incidence among non-Hispanic Black and other racial groups, survival outcomes improved equally in all races when irAEs occurred (82).

The correlation between gender and irAEs in research is inconsistent. Two recent meta-analysis showed no significant differences between genders in the incidence, severity, or hospitalization rates due to irAEs (83, 84). However, many other real-world studies indicate that female patients may be more prone to developing and experiencing severe irAEs, identifying gender as an independent risk factor (85–87).

Numerous studies have shown that the incidence of any-grade irAEs is correlated with better clinical outcomes, including PFS and OS. Moreover, patients who experienced multi-system irAEs had better PFS and OS compared to those with single-system or no irAEs (88–95). However, this correlation is not always consistent or reproducible in some other literature. Several high-quality studies have found neutral (96, 97) or even negative impacts (98, 99) of irAEs on survival, highlighting conflicting results in the literature.

Interestingly, our analysis showed that patients with irAEs had worse survival outcomes at the 30-day landmark analysis (mPFS of 3.3 vs. 7.1 months, p = 0.0085; mOS of 4.4 vs. 9.0 months, p = 0.0004), deviating from studies that reported improved outcomes in patients who developed irAEs. Haratani et al. found better PFS and OS among NSCLC patients treated with nivolumab who developed irAEs (mPFS of 10.1 vs. 3.7 months and mOS of 24.5 vs. 11.2 months) (100).

The relationship between the timing of irAEs onset and patient survival remains a subject of considerable debate. A meta-analysis by Huang Y et al. in patients with advanced or recurrent lung cancer suggested that early-onset irAEs were associated with worse outcomes, including a higher risk of disease progression (HR = 2.16; 95% CI: 1.62–2.89; *P* < 0.001) and increased mortality (HR = 2.63; 95% CI: 1.93–3.59; *P* < 0.001) (101). Similarly, a large retrospective analysis of 101,451 patients from the TriNetX database by Sayer & Ozaki (2024) found that early-onset colitis or pneumonitis was significantly associated with poorer 1-year survival compared to patients with no irAEs (102).

It is crucial to note that many studies exploring the association between irAEs and survival have been criticized for not properly accounting for immortal time bias (103). This bias occurs because patients must survive long enough to develop an irAE, which can lead to a spurious association between irAE occurrence and improved survival outcomes. This analytical flaw can confound the true relationship (103). To mitigate this bias, methodological approaches such as landmark analysis and time-dependent Cox regression are necessary. Using a landmark analysis, Kfoury et al. found no significant difference in overall survival (OS) or progression-free survival (PFS) between patients who developed an irAE within the first 12 weeks landmark of therapy and those who did not (104).

Furthermore, in our own analysis, we applied both an adjusted landmark analysis and a time-dependent Cox model to rigorously evaluate this association while accounting for potential confounders (e.g., age, ECOG PS, comorbidities, tumor burden, and cancer type). Our findings consistently indicate that the occurrence of irAEs is associated with poorer survival outcomes. Specifically, irAEs were linked to worse OS at the 30-day landmark when applying adjusted landmark analysis (HR 2.13, 95% CI 1.34–3.3, *P* = 0.001) and worse PFS (HR 1.88, 95% CI 1.22–2.87, *P* = 0.004).

This association was further supported by our time-dependent Cox analysis, which also demonstrated a link between irAEs and worse OS (HR 1.86, 95% CI 1.23–2.79, *P* = 0.003) and PFS (HR 1.96, 95% CI 1.41–2.72, *P* = 0.001).

Our findings may reflect the uneven impact of severe or fatal irAEs on decreased survival, particularly pulmonary and cardiac toxicities, which are associated with early mortality (100).

Some reports linked the adverse prognostic association of early irAEs to the early treatment discontinuation and the requirement for systemic high dose corticosteroids, both of which may attenuate the therapeutic efficacy of ICIs (105). Moreover, some literature showed that rapid clinical deterioration observed in this context may in certain instances represent hyperprogressive disease rather than bona fide immune toxicity, a phenomenon independently associated with markedly poor prognosis (106).

Risk factors associated with irAEs in our study included lower platelet counts (p = 0.015 at baseline; p = 0.012 at week 6) and higher baseline TSH levels (p = 0.048). Similarly, previous studies have identified thrombocytopenia and elevated TSH as potential biomarkers for irAEs underscoring their role in risk stratification (66, 107, 108).

Studies assessing the association between age and irAEs incidence introduce another layer of uncertainty. While some studies reported no correlation or reduced risk (109, 110), others showed increased risk of toxicity in older adults (111, 112).

Notably, pulmonary toxicity was ranked first among older patients, while hepatic toxicity was less frequent (113). On the contrary, real-world data noted increased irAEs in younger individuals (114). Our analysis revealed that older adults (≥65 years) experienced a marginally higher, though non-significant, irAEs rate (62.7% vs. 53.3%, p = 0.19). However, dermatologic toxicities were significantly more frequent in older patients (17.9% vs. 7.1%, p = 0.018).

Interestingly, irAEs incidence was similar in patients with good (ECOG 0–1) and poor (ECOG ≥2) PS (55.2% vs. 57.8%, p = 0.77), contrasting with prospective trials that often exclude ECOG ≥2 patients and usually associated with poor prognosis (115).

Moreover, we showed that cardiac irAEs were significantly more common in patients with poor PS (10.9% vs. 1.7%, p = 0.036) (116), consistent with evidence of increased vulnerability in frail patients (117–119). Salem et al., using FDA pharmacovigilance data, showed that cardiovascular toxicities -particularly myocarditis- had disproportionately high fatality (~50%) in patients with frailty or comorbidities (50). Similarly, Hu et al. linked poor functional status and baseline cardiovascular comorbidities with increased cardiac irAEs risk (120).

In our analysis, we found that rechallenging ICIs after the development of high-grade irAEs was associated with a risk of recurrence. Hepatic, dermatologic, and endocrine irAEs recurred in 9%, 4%, and 2% of cases, respectively. Matching the finding from some current literature, where immune related hepatitis, pancreatitis, pneumonitis, and nephritis frequently recurred upon rechallenge with PD1 inhibitors in patients who previously experienced severe irAEs with dual PD-1 and CTLA-4 blockade (121).

This study has several strengths and some limitations. Firstly, our study represents one of the largest real-world cohorts on ICIs use from the Middle East, a region that is often unrepresented in landmark clinical trials. To decrease reporting or misclassification biases, we used CTCAE V5.0 as an objective tool to stratify irAEs grades. Moreover, to account for immortal time bias, we applied adjusted landmark analysis of survival outcomes at different points: 30, 60, 90, 180 and 360 days from the start of ICIs therapy Additionally, we performed time-dependent Cox analysis to account for the dynamic occurrence of irAEs.

We acknowledge some limitations. Firstly, the retrospective design may have led to misreporting of some irAEs, particularly lower-grade or late-onset events.

Secondly, although a multidisciplinary review was conducted, provenance of death to immune-related toxicity in the absence of biopsy or autopsy remains challenging and may lead to over- or under-estimation of fatal irAEs rates. While the study was conducted at a single tertiary center, it is important to note that NCCCR is the only adult cancer center in the State of Qatar, receiving all national referrals from primary and private health sectors. This provides a comprehensive, population-based dataset; however, differences in treatment practices, supportive care, or referral patterns across other regions or healthcare systems may limit the generalizability of our findings.

## Conclusion

In this real-world, population-based cohort from Qatar, irAEs were frequent, clinically diverse, and contributed to significant early morbidity and mortality, particularly in the setting of fatal pulmonary, cardiac, hepatic, and renal toxicities. Our data demonstrates that irAEs occurred across a broad temporal spectrum, including hyperacute and delayed forms, underscoring the need for ongoing monitoring throughout and after treatment. The higher rate of fatal irAEs, especially among older or frail patients, emphasizes the importance of pre-treatment risk stratification and close post-treatment surveillance.

Using adjusted landmark and time-dependent Cox analyses, early-onset irAEs were associated with poorer survival outcomes, in contrast to previous reports.

Patients with poor baseline PS were more likely to develop cardiac irAEs, while older adults (more than or equal to 65 years old) showed an increased risk of dermatologic irAEs.

A higher number of ICI treatment cycles remained the only independent predictor of irAEs in the multivariable logistic regression analysis, whereas thrombocytopenia and elevated TSH emerged as potential risk markers in univariate analysis.

These findings reinforce the need for careful baseline assessment, timely recognition, and multidisciplinary management of irAEs to optimize ICI safety and effectiveness.

Further prospective, multi-institutional studies are warranted to validate these associations and refine predictive tools for risk stratification and outcomes.

## Data Availability

The datasets presented in this article are not readily available because the data presented in this study are available on request from the corresponding author. the data is not publicly available due to privacy or ethical restrictions. requests to access the datasets should be directed to nomar4@hamad.qa.
