# Correction: Safety of immune checkpoint inhibitors for cancer treatment: real-world retrospective data analysis from Qatar (SAFE-ICI-Q study)

**DOI:** 10.3389/fimmu.2026.1902412

**Published:** 2026-06-19

**Authors:** Nabil E. Omar, Shereen Elazzazy, Anas Hamad, Mohamed Omar Saad, Aya Alasmar, Sahar M. Nasser, Maria Benkhadra, Hebatalla M. Afifi, Farah I. Jibril, Rawan A. Dawoud, Mohamed S. Hamid, Afnan Alnajjar, Arwa O. Sahal, Amaal Gulied, Hazem Elewa

**Affiliations:** 1 Pharmacy Department, National Center for Cancer Care and Research, Hamad Medical Corporation, Doha, Qatar; 2 Clinical and Population Health Research Program, College of Pharmacy, QU Health, Qatar University, Doha, Qatar; 3 College of Pharmacy, QU Health, Qatar University, Doha, Qatar; 4 Pharmacy Department, Al Wakra Hospital, Hamad Medical Corporation, Doha, Qatar; 5 Medical Oncology Department, National Center for Cancer Care and Research, Hamad Medical Corporation, Doha, Qatar

**Keywords:** immune checkpoint inhibitors, immune-related adverse events, real-world data, cancer immunotherapy, biomarkers, progression-free survival, overall survival

In the **Acknowledgements** and the **Funding** statement, this sentence was erroneously omitted: “The publication of this article was funded by the Qatar National Library.”

The original version of this article has been updated.

