# Supplementary material for: Safety of immune checkpoint inhibitors for cancer treatment: real-world retrospective data analysis from Qatar (SAFE-ICI-Q study)
Source: Front Immunol. 2025 Oct 29;16:1665716. doi: 10.3389/fimmu.2025.1665716 (PMC12605147; doi:10.3389/fimmu.2025.1665716)
Supplement: Supplementary file 1 [file SupplementaryFile1.pdf]

Supplementary tables:

Table S1: **Distribution of irAEs per system**

Table S1a

Endocrine immune Adverse Event (irAEs) 47 (26.4 %)	Description	Number of Events	Percentage out of total irAEs (%)
	Hypothyroidism	29	16.3%
	Adrenal insufficiency	5	2.8%
	Hyperthyroidism	3	1.7%
	Electrolyte imbalance*	4	2.2%
	Hyperglycemia	2	1.1%
	New DM onset	2	1.1%
	Diabetic ketoacidosis	2	1.1%
	* Hyponatremia, hyperkalemia, hypercalcemia, and hypomagnesemia		

Table S1b

Gastro-intestinal irAEs 12 (6.7%)	Description	Number of Events	Percentage out of total irAEs (%)
	Diarrhea	4	2.2%
	Persistent vomiting & GASTRITIS	3	1.7%
	Pancreatitis	3	1.7%
	Colitis	2	1.1%

Table S1c

Cardiac irAEs 10 (5.6%)	Description	Number of Events	Percentage out of total irAEs (%)
	Myocarditis	5	2.8%
	Edema*	2	1.1%
	Drop in Ejection Fraction	1	0.6%
	Massive pericardial effusion	1	0.6%
	Cardiac arrest	1	0.6%
	* One event of Severe facial edema and eye swelling , another event of face and neck edema		

Table S1d

Hepatic irAEs 22 (12.4%)	Description	Number of Events	Percentage out of total ICPI agents (%)
	Transaminitis	17	9.6%
	Hyperbilirubinemia	2	1.1%
	Hyperammonemia and Coagulopathy	2	1.1%
	Cholangitis	1	0.6%

Table S1e

Hematological irAEs 15 (8.4%)	Description	Number of Events	Percentage out of total ICPI agents (%)
	Thrombocytopenia	5	2.8%
	Anemia	4	2.2%
	Pancytopenia	2	1.1%
	Bicytopenia	1	0.6%
	Gingival bleeding	1	0.6%
	Eosinophilia	1	0.6%
	Neutropenia	1	0.6%

Table S1f

Neurological irAEs 9 (5.1%)	Description	Number of Events	Percentage out of total ICPI agents (%)
	Fatigue	5	2.8%
	Neuropathy	2	1.1%
	Limbic encephalopathy	1	0.6%
	Insomnia	1	0.6%

Table S1g

Other irAEs 23 (12.9%)	Description	Number of Events	Percentage out of total ICPI agents (%)
	Musculoskeletal	9	5.1%
	Renal	8	4.5%
	Infusion reaction	3	1.7%
	Infectious	2	1.1%
	Ocular	1	0.6%

Table S2 : **Time of onset of irAEs**

**Time of onset of irAEs [days (IQR)]**	**Skin**	**Endocrinopathies**	**Pulmonary**	**Gastrointestinal**	**Cardiac**	**Hepatic**	**hematologic**	**Neurologic**	**Other #***
	81 (14 – 192.5)	106 (55 – 232)	53 (25 – 229)	55 (11.5 – 102.5)	68 (27 –114)	42 (20 – 102)	53 (5 – 591	77 (13 – 251)	55 (1 – 376)

Other irAEs: Musculoskeletal, Renal, Infusion reaction, Infectious and Ocular irAEs
* Three patients developed infusion reaction after 10 minutes of first infusion

Table S3 : **Management of irAEs.**

irAEs management		**Skin**	**Endo**	**Pulmonary**	**GI**	**cardiac**	**Hepatic**	**hem**	**Neuro**	**Other***
Immunosuppressive agents	Steroids	7	5	10	2	4	7	1	3	10
	Non-steroids	0		0	0	0	9	0	0	3
Non immunosuppressive agents	Other management	17	42	6	10	6	6	14	6	15

**Counts are management episodes and are not mutually exclusive, as some patients received >1 modality.*

Table S4: **Characteristics of Fatal immune related adverse effects**

**System affected with the fatal irAEs**	**Number of irAEs n= 20 (%)**	**Time to death from fatal irAEs, median days**
Hepatic	4 (20)	20.4
Pulmonary	5 (25)	6
Cardiac	4 (20)	5.7
Renal	4 (20)	18.8
Endocrine	1 (5)	9.7
Nervous system	1 (5)	9
Hematological	1 (5)	9

Table S5: **Time of onset of fatal irAEs**

**Time of onset of fatal irAEs**	**Number of irAEs n= 20 (%)**
Hyper-acute (very early): occur from Day 1 after initiation of ICIs to 21 days)	14 (70)
Acute (early): occur > 21 days to less than 180 days	6 (30)

Table S6: **Adjusted Landmark Analysis of OS and PFS**

Landmark (days)	OS HR (95% CI)	OS P value	PFS HR (95% CI)	PFS P value
30	2.13 (1.34–3.37)	0.001	1.88 (1.23–2.88)	0.004
60	1.28 (0.82–2.01)	0.272	1.34 (0.90–1.99)	0.147
90	1.26 (0.78–2.02)	0.346	1.25 (0.82–1.90)	0.307
180	1.33 (0.74–2.38)	0.345	1.09 (0.66–1.81)	0.729
360	2.67 (0.88–8.10)	0.083	1.16 (0.55–2.43)	0.695

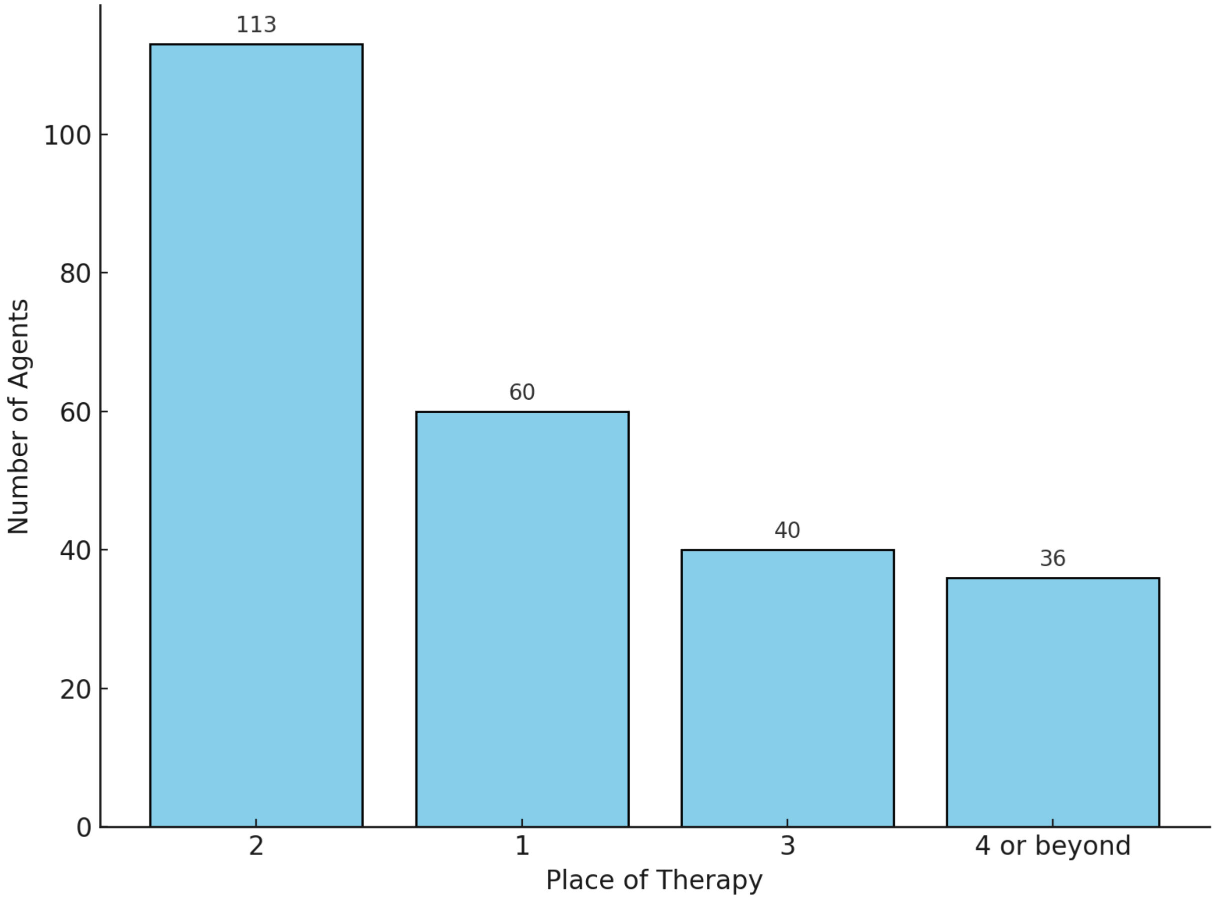

Fig S1: Distribution of place of therapy of ICI agents.

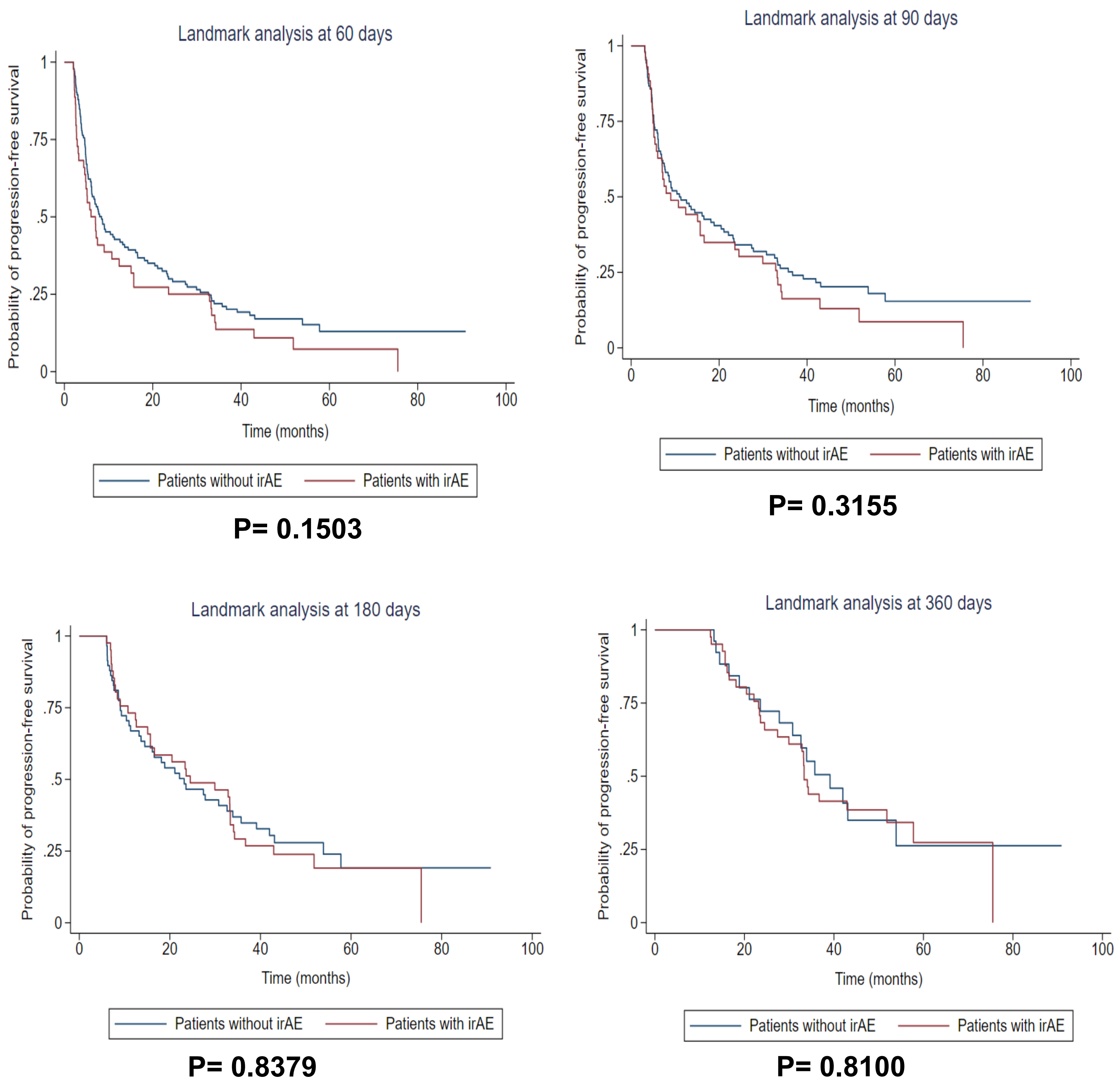

Fig S2A: Unadjusted landmark analysis of PFS at 60,90,180 and 360 days

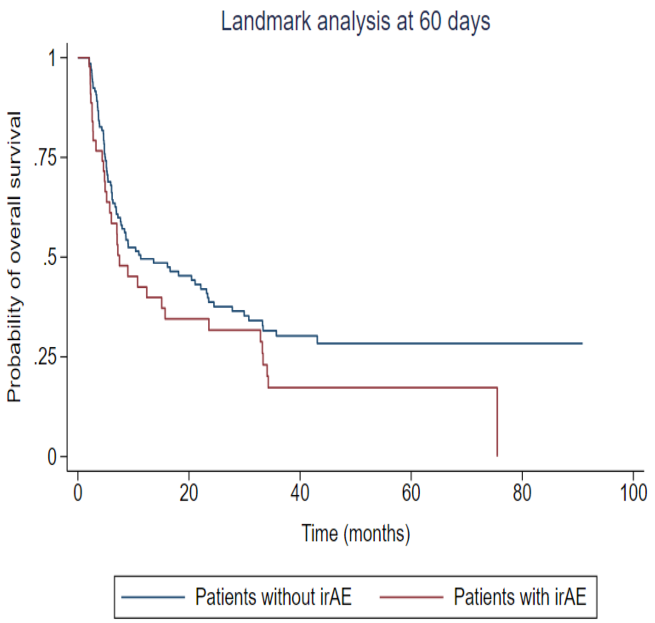

P= 0.1073

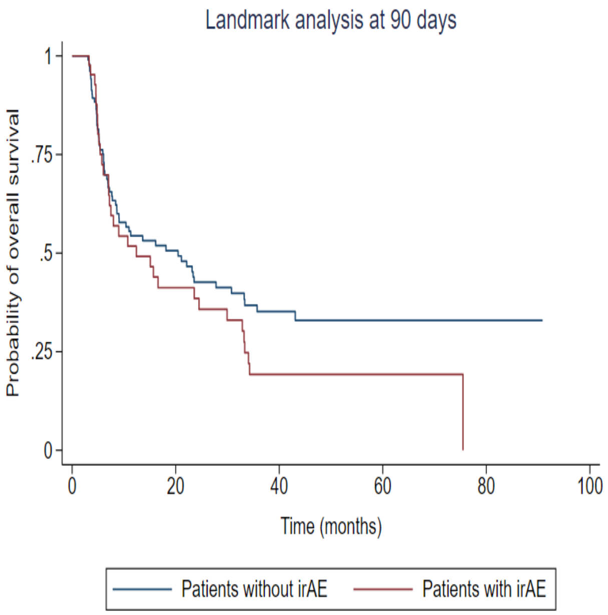

P= 0.2009

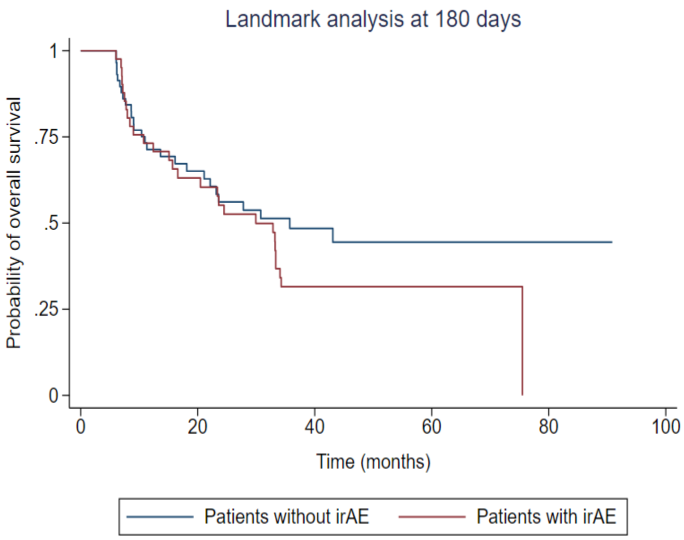

P= 0.2905

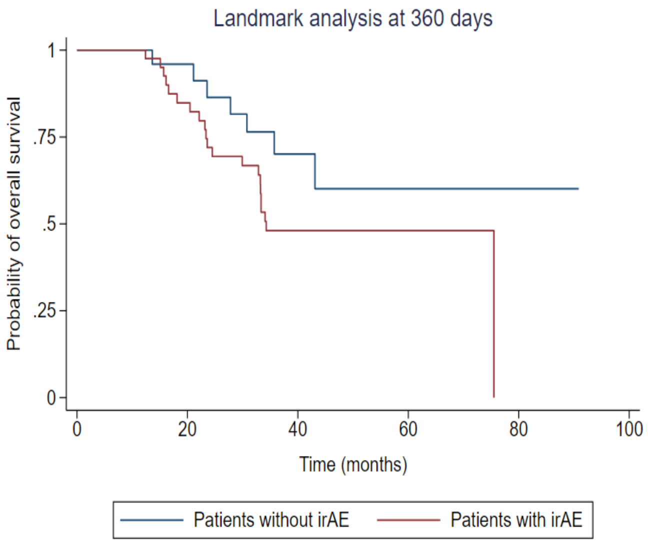

P= 0.1470

Fig S2B: Unadjusted landmark analysis of OS at 60,90,180 and 360 days
